# Chronic intermittent ethanol vapor exposure alters local c‐Fos and functional connectivity patterns evoked by alcohol cues

**DOI:** 10.1111/acer.70258

**Published:** 2026-02-19

**Authors:** Ankit Sood, Filip Hanak, Antony Joseph, M. J. Carpio, Runbo Gao, Adem Selimovic, Seth D. C. Weir, Fleur Uittenbogaard, Diana Augustin, Jocelyn M. Richard

**Affiliations:** ^1^ Department of Neuroscience University of Minnesota Minneapolis Minnesota USA; ^2^ Medical Discovery Team on Addiction University of Minnesota Minneapolis Minnesota USA; ^3^ Graduate Program in Neuroscience University of Minnesota Minneapolis Minnesota USA

**Keywords:** alcohol cues, ethanol vapor, hippocampus, nucleus accumbens, prefrontal cortex

## Abstract

**Background:**

Chronic intermittent ethanol (CIE) vapor inhalation is a widely used model for studying alcohol dependence. Additionally, cues paired with ethanol can drive and invigorate seeking behavior. However, the impact of ethanol‐paired cues on neural activation following a history of CIE vapor exposure remains poorly understood. Here, we examined the impact of ethanol‐paired cues in rats with or without a history of CIE vapor exposure on neuronal activation in the medial prefrontal cortex (mPFC), nucleus accumbens (NAc), and the hippocampus (HPC) using c‐Fos as a marker.

**Methods:**

Male and female Long–Evans rats with a history of air or CIE vapor exposure were exposed to previously learned alcohol‐associated cues under extinction conditions. Rats were perfused either immediately following cue exposure or with a delay to allow the development of c‐Fos protein. c‐Fos expression levels were analyzed in the mPFC, NAc, and HPC.

**Results:**

CIE vapor exposure led to a region‐specific effect on cue‐evoked c‐Fos levels, with enhanced cue‐evoked c‐Fos levels observed in the prelimbic mPFC of CIE rats. Correlation analysis of c‐Fos expression across brain regions revealed that CIE vapor exposure also led to altered functional connectivity patterns.

**Conclusions:**

Our results suggest that CIE vapor exposure affects cue‐evoked c‐Fos expression in the prelimbic mPFC as well as influencing functional connectivity across regions.

## Graphical abstract

How chronic intermittent ethanol (CIE) vapor exposure impacts alcohol cue‐evoked responses is not clear. We analyzed c‐Fos expression in the mPFC, NAc, and the hippocampus in CIE‐exposed rats perfused at different time points. CIE vapor‐exposed rats showed altered c‐Fos levels only in the PL mPFC, and both CIE and perfusion timing had an impact on functional connectivity across regions. Our results suggest that previous ethanol exposure can affect subsequent ethanol cue‐evoked neural activation and functional connectivity patterns.

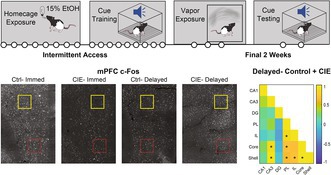

## INTRODUCTION

Chronic intermittent exposure (CIE) to ethanol vapor is a model of alcohol dependence that is widely used in rodents (Becker & Lopez, 2004; Gilpin et al., 2008; Vendruscolo & Roberts, 2014). This model results in elevated alcohol consumption, self‐administration, and increased blood and brain ethanol levels (Becker & Lopez, 2004; Gilpin et al., 2008, 2009; Vendruscolo & Roberts, 2014), especially in male rodents (Becker & Lopez, 2004; Finn et al., 2007; Griffin, Lope,z, Yanke, et al., 2009; Griffin, Lopez, & Becker, 2009; Lopez et al., 2011; McCool & Chappell, 2015). Cues associated with alcohol availability and consumption are another factor that can independently potentiate alcohol‐seeking behaviors and reinstatement following abstinence (Jupp et al., 2011; Millan et al., 2015; Remedios et al., 2014); however, limited work has been conducted on how CIE alters the impact of alcohol cues on behavior and the brain. Studies have reported an increase in operant self‐administration of alcohol in response to cues previously associated with alcohol consumption post CIE vapor exposure (Gass et al., 2017; O'Dell et al., 2004). CIE is also known to modulate cue‐induced reinstatement of alcohol seeking, depending upon the presence of alcohol during the acute withdrawal period. Studies where alcohol was present during the acute CIE withdrawal period have demonstrated enhanced cue‐induced reinstatement compared with reports where alcohol was not present during the acute withdrawal (Ciccocioppo et al., 2003; Liu & Weiss, 2002, 2003). Previously, we investigated the effects of CIE on cue‐elicited alcohol seeking in rats by performing the cue‐alcohol pairing before or after the CIE (Carpio et al., 2022). We observed that CIE enhanced responsivity to cues previously paired with alcohol only when the animals had access to alcohol during acute withdrawal.

While the prior work suggests that CIE can alter behavioral responses to alcohol, the neurobiological mechanisms underlying these effects remain unknown. Previous studies have investigated the effects of CIE vapor exposure on c‐Fos expression as a proxy for neural activation at various withdrawal time points and after re‐exposure to ethanol (Kimbrough et al., 2020; Roland et al., 2023; Smith et al., 2020; Vilpoux et al., 2009). However, while there is ample evidence about the behavioral and neural changes in models of CIE vapor exposure and withdrawal, much less is known about the effect of CIE vapor exposure coupled with previously learned alcohol cues on brain activation patterns. In this study, we analyzed neural activation patterns in animals that underwent alcohol‐cue training before CIE vapor exposure and subsequent behavioral testing as described in a previously published report (Carpio et al., 2022). Rats were exposed to previously trained alcohol cues under extinction conditions, followed by perfusion either immediately or with a delay of 60–90 min for c‐Fos protein expression analysis in the medial prefrontal cortex (mPFC), nucleus accumbens (NAc), and the hippocampus. These regions were chosen because of previous evidence indicating that they are responsive to both CIE vapor exposure and ethanol‐paired cues. In a previously published report, Smith et al. (2020) showed that the mPFC and NAc display dynamic c‐Fos expression patterns in response to CIE and at various withdrawal time points post CIE vapor exposure. Both the mPFC and NAc are activated by ethanol‐paired cues in cue‐induced reinstatement paradigms following extinction (Jupp et al., 2011; Zhao et al., 2006). Additionally, distinct roles for the prelimbic (PL) and infralimbic (IL) mPFC and core and shell of the NAc have been shown for cue‐ and context‐mediated reinstatement of alcohol seeking (Chaudhri et al., 2010; Groblewski et al., 2012; Willcocks & McNally, 2013). The hippocampus has been shown to display altered c‐Fos expression in response to CIE vapor exposure (Roland et al., 2023; Smith et al., 2020) and ethanol‐predictive cues (Dayas et al., 2007; Zhao et al., 2006). To assess the impact of a history of CIE vapor exposure on functional connectivity, we examined correlations between c‐Fos expression patterns across brain regions.

## MATERIALS AND METHODS

### Subjects

Animals used in this study (male and female Long–Evans rats, Envigo) were from experiment 1 in a previously published report from our lab (Carpio et al., 2022). Rats were individually housed with ad libitum access to food (Teklad Global 18% Protein Rodent Diet, #2018, Envigo) and water and maintained on a 14 h–10 h light–dark cycle (lights on at 6 am). Additional details about behavioral procedures and results can be found in the published manuscript (Carpio et al., 2022). Procedures relevant to this study are described briefly in the following sections. All experimental procedures were approved by the Institutional Animal Care and Use Committee at the University of Minnesota and carried out in accordance with the Guide for the Care and Use of Laboratory Animals (NIH).

### Homecage drinking and discriminative stimulus (DS) task training

All rats were exposed to 8 weeks of intermittent access to 15% ethanol as described previously to acclimate them to the taste and pharmacological properties of ethanol and to ensure that they were motivated to learn the DS task for the reward (Carpio et al., 2022).

DS task training occurred in operant chambers equipped with a speaker, white noise and tone generators and a reward port with a head entry detector (Med Associates Inc., Fairfax, VT). Before training in the DS task, all animals underwent two to three magazine training sessions which consisted of 30 reward deliveries (0.1 mL 15% ethanol). Each session lasted until the animal entered the port after 30 successive reward deliveries or 120 min elapsed, whichever occurred earlier, and animals repeated magazine training if they failed to collect all the rewards. After magazine training, rats underwent DS task training as described elsewhere (Ottenheimer et al., 2019; Richard et al., 2016, 2018). Briefly, if rats made a port entry during an auditory cue (DS; a combination of 2700‐ and 4100‐kHz tones), they earned a 15% ethanol reward (0.1 mL). Port entries during a control auditory cue (neutral stimulus or NS; white noise) or outside of the cue period had no consequences. Training comprised five stages. In stages 1–4, 30 DS cues were presented with decreasing lengths (60 s, 30 s, 20 s, and 10 s). Starting from stage 1, rats advanced to the next stage if they made port entries on at least 60% DS cues. Stage 5 included the introduction of the 10s NS cue presented 30 times for a total of 60 cues. All cues in all stages were presented with variable inter‐trial intervals and sessions lasted 90 min. During stage 5, DS and NS cues were presented in a pseudorandom order.

### Chronic Intermittent Ethanol (CIE) Vapor Exposure

Following training on the DS task, rats underwent chronic intermittent exposure (CIE) to ethanol vapor in vapor inhalation chambers (La Jolla Alcohol Research, Inc., San Diego, CA) as described previously (Carpio et al., 2022). Rats were divided into control (air) or CIE groups. Vapor exposure was carried out by placing the rats in their home cages inside the inhalation chambers and passing ethanol vapor (95%) through the chambers. Each round consisted of 14 h of vapor exposure followed by 10 h of withdrawal and was repeated for four sequential nights a week, for 3 weeks. Approximately 2.5 weeks post CIE, rats underwent a cue test under extinction conditions followed by retraining with ethanol reward as described previously (Carpio et al., 2022). The first cue test consisted of presentations of 10 probe cues (5 DS and 5 NS) under extinction conditions (no ethanol reward). This was followed immediately by presentation of 60 cues (30 DS and 30 NS) under retraining conditions, with a port entry during the DS once again resulting in an alcohol reward. During the next 48 h, rats underwent three additional testing sessions, with increasing numbers of trials or amounts of ethanol delivery, as described previously (Carpio et al., 2022). Blood ethanol concentrations were measured during the CIE period as described previously (Carpio et al., 2022).

### Final cue test

Following the last retraining session, rats underwent a final cue probe test under extinction conditions (no EtOH reward). The test lasted 30 min and consisted of 10 DS and 10 NS presentations in a randomized fashion without any reward delivery. Testing under extinction conditions allowed us to examine cue‐driven behavioral and neural responses.

### Brain collection and c‐Fos immunohistochemistry

Rats were perfused either immediately upon completion of the cue probe test (immediate) or with a delay of 60–90 min (delayed). Since c‐Fos protein expression peaks 60–90 min of after stimuli that activate cells (Kovács, 2008; Lara Aparicio et al., 2022), this design allowed us to differentiate Fos induction as a result of the final behavioral test from putative baseline Fos levels. For perfusion, rats were deeply anesthetized with pentobarbital (i.p.) and then transcardially perfused with phosphate‐buffered saline (PBS), followed by 4% paraformaldehyde (PFA). Brains were collected and postfixed in 4% PFA at 4°C for 24 h and then placed in 20% sucrose in PBS until they sank to the bottom. Brains were then stored in a cryoprotectant solution at −20°C till sectioning. 40‐μm sections were cut on a microtome and collected in PBS before further processing. c‐Fos immunohistochemistry was carried out on the free‐floating sections. Sections were first incubated with the blocking solution (10% normal goat serum in PBS with 0.3% Triton X‐100 (PBTx)) for 2 h at room temperature. Following blocking, sections were incubated overnight at room temperature with the primary antibody (rabbit anti c‐Fos (9F6); 1:3000, Cell Signaling Technology, Cat# 2250) in PBTx. Sections were then washed in 1× PBS (3 washes, 15 min each) before incubating with the secondary antibody (Donkey anti‐Rabbit Alexa Fluor‐555; 1:250; ThermoFisher, Cat# A31572) for 3 h. Sections then underwent three PBS washes (1× PBS, 15 min each) before being mounted on Superfrost Plus slides and coverslipped using Vectashield Hardset with DAPI (Vector Laboratories, CA).

### Image analysis

Images were captured at 20× magnification using a Keyence BZ‐X710 microscope (Keyence, Itasca, IL) and a monochromatic camera. c‐Fos‐positive cells were quantified using ImageJ (NIH). Regions of interest (ROIs) were drawn and c‐Fos‐positive cells were quantified within the ROIs based on size, circularity, and intensity cutoffs. A final c‐Fos nuclei density (c‐Fos counts per mm^2^) was obtained for each ROI by dividing the c‐Fos count by the area of the ROI for each hemisphere. Quantification was done by an experimenter blind to the experimental groups. The brain regions analyzed were the infralimbic and prelimbic mPFC (AP 3.0–2.76 mm; 1–3 slices per animal); NAc core and medial shell (AP 2.28–2.18 mm; 2 slices per animal); and the CA1, CA3, and dentate gyrus (DG) regions of the HPC (AP ‐2.76 mm to −3.12 mm; 2–3 slices per animal).

### Statistical analysis

Data analysis was conducted using MATLAB (Mathworks) and R (The R Project). Behavioral and c‐Fos expression data were analyzed using linear mixed‐effects models (LME) followed by ANOVA with statistical significance set at *p* < 0.05. Effect sizes (eta squared) were calculated using the measures of effect size (MES) toolbox in MATLAB (Harald Hentschke, 2025) and reported wherever we observed a significant ANOVA main effect or interaction effect. Fixed effects for CIE vapor exposure and perfusion, along with a random effect for subjects, were included in the LME analysis. Since we were underpowered to assess c‐Fos expression in males and females independently and model comparisons indicated that adding sex as a factor did not improve each model data were analyzed pooled across males and females, without sex as a factor. To assess the relationship between c‐Fos expression and behavioral performance on the final test, LME analysis was done with port entry probability as the response variable and c‐Fos count as the fixed effect along with a random effect for subjects. The data were plotted using a generalized linear model (GLM) fit function in the gramm package in MATLAB. For the inter‐regional c‐Fos correlation matrix, Pearson's correlation coefficient (*R*) was calculated for each pair of regions, and the *R* values were used to plot the correlation matrix. Pairwise post hoc comparisons with Tukey's correction were computed in *R*.

## RESULTS

### Prior CIE vapor inhalation did not affect behavioral performance to ethanol cues under extinction conditions

**FIGURE 1:**
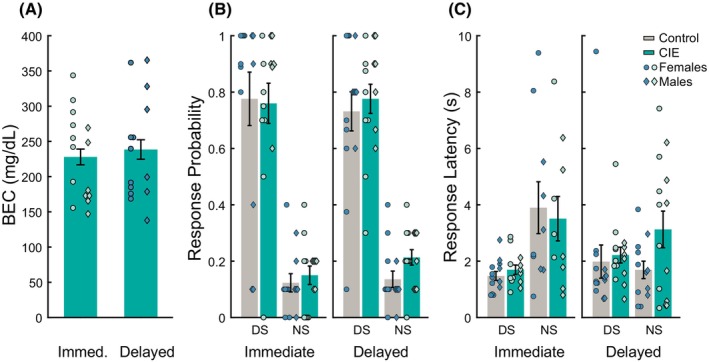
Final DS task under extinction conditions. Rats (*n* = 27 control (12 males); 29 CIE (13 males)) underwent a probe test under extinction conditions before perfusions. (A) Blood ethanol concentrations measured during CIE exposure. (B) Probability of entering the port in response to the DS or NS cues in rats exposed to air (control) or CIE vapor (CIE) after DS task training. (C) Latency to enter the port in response to DS or NS cues in control or CIE vapor‐exposed rats. Symbols represent individual animals (circles: Females, diamonds: Males).

In a previously published report, we found that CIE enhanced behavioral response to a previously trained alcohol DS, but only in rats that received access to oral alcohol during acute withdrawal during the CIE phase of the experiment (Carpio et al., 2022). In this study, we examined c‐Fos expression in the same cohort of rats that received access to oral alcohol during acute withdrawal. Following initial testing for cue responses under extinction conditions, rats underwent retraining with the DS and NS cues, where port entry during the DS resulted in ethanol reward delivery. Rats were then administered a final cue probe test under extinction conditions before being perfused immediately or with a delay of 60–90 min. The final test allowed us to assess behavioral responses to alcohol cues in the absence of ethanol reward after retraining and to examine cue‐evoked neural activity. Analysis of blood ethanol concentrations (BECs) during the CIE exposure phase revealed no significant differences between rats that were assigned to be perfused immediately or with a delay (Figure 1A, main effect of perfusion timing: *F*(1, 25) = 0.86, *p* = 0.36; main effect of sex: *F*(1, 25) = 4.02, *p* = 0.06, interaction between perfusion timing and sex: *F*(1, 25) = 3.44, *p* = 0.08) indicating that rats in both cohorts were exposed to similar levels of ethanol. When we analyzed the behavioral performance, we observed significant main effects of sex and cue on port entry probability (Figure 1B; sex: *F*(1, 104) = 5.065, *p* < 0.05; cue: *F*(1,104) = 56.32, *p* < 0.0001) and only an effect of cue on port entry latency (Figure 1C; *F*(1,93) = 5.87, *p* < 0.05). We also observed a significant interaction between CIE and sex for port entry probability (*F*(1,104) = 6.86, *p* < 0.05). While we did not find any significant pairwise comparisons to explain this interaction, it appeared to be driven by an increase in port entry probability in CIE males, but not CIE females. We did not observe any significant interactions between sex and cue (*F*(1,104) = 2.42, *p* = 0.12); CIE and cue (*F*(1,104) = 2.25, *p* = 0.13); or CIE, sex, and cue (*F*(1,104) = 2.79, *p* = 0.09). No significant interactions between CIE, cue, and sex were noted for port entry latency (*F* values from 0.003 to 0.573, *p* values from 0.45 to 0.95). Overall, CIE vapor inhalation did not significantly affect behavioral responses to ethanol‐paired cues during the final test under extinction conditions, which contrasted with our findings from the earlier extinction test in these same animals, where we found that CIE rats displayed increased responding to DS cues (Carpio et al., 2022). This change could be explained by the impact of increased time since CIE ethanol exposure, additional experience with testing under extinction conditions or other factors.

### CIE vapor inhalation and c‐Fos reactivity across multiple brain regions

To assess the effect of CIE vapor inhalation on cue‐evoked neural activation patterns, we compared levels of c‐Fos‐positive cells in the medial prefrontal cortex (mPFC), nucleus accumbens (NAc), and the hippocampus (HPC) of rats perfused either immediately or with a 60–90 min delay following the final test. c‐Fos expression was quantified in the functional and anatomical subdivisions of the regions: the prelimbic (PL) and infralimbic (IL) mPFC; NAc core and medial shell; and the CA3, CA1, and DG subdivisions of the hippocampus. Since after splitting the subjects by vapor exposure (control and CIE) and perfusion timing (immediate and delayed) group, we were underpowered to analyze males and females separately c‐Fos expression analysis was done with subjects pooled across sexes.

### CIE enhances cue‐evoked c‐Fos in the prelimbic medial prefrontal cortex

**FIGURE 2:**
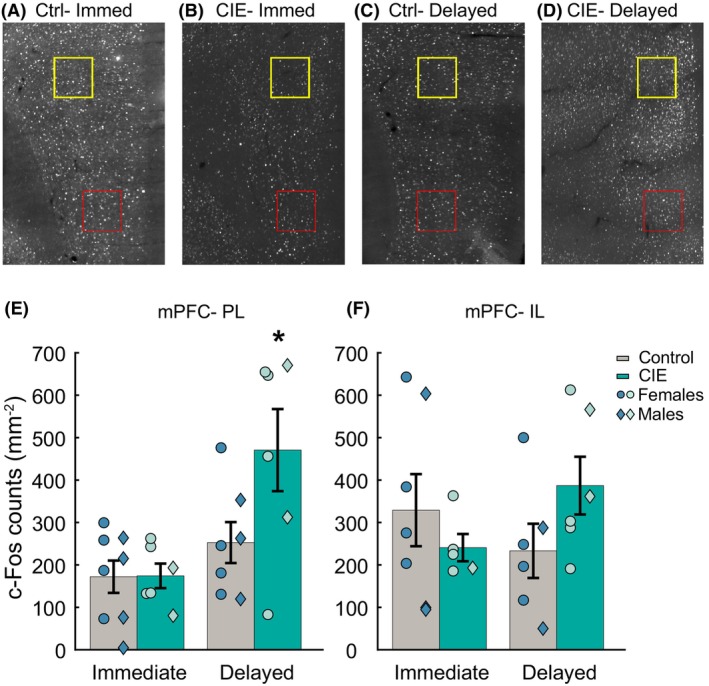
Effect of CIE vapor exposure on cue‐evoked c‐Fos in the mPFC. Rats (PL mPFC: Immediate control: *N* = 8 (4 males), immediate CIE: *N* = 6 (2 males), delayed control: *N* = 7 (3 males), delayed CIE: *N* = 6 (2 males); IL mPFC: Immediate control: *N* = 7 (3 males), immediate CIE: *N* = 5 (1 male), delayed control: *N* = 6 (2 males), delayed CIE: *N* = 6 (2 males), 1–3 brain slices per animal) were perfused either immediately after the final behavioral test or with a delay of 60–90 min to analyze cue‐evoked c‐Fos expression. (A–D) Representative c‐Fos images, boxed region indicates the region of interest (ROI) used for c‐Fos quantification (PL in yellow and IL in red). (E) Rats in the delayed CIE vapor exposure group had increased c‐Fos expression in the prelimbic (PL) mPFC. (F) No difference in c‐Fos expression was observed in the infralimbic (IL) mPFC. Data represented as mean ± SEM. Circles represent individual rats. *p < 0.05 compared to the immediate‐CIE group. Symbols represent individual animals (circles: Females, diamonds: Males).

Within the mPFC, our analysis revealed a significant main effect of CIE (*F*(1,61) = 9.363, *p* = 0.003, eta = 0.08, *p* = 0.01), perfusion time (*F*(1,61) = 15.63, *p* < 0.005, eta = 0.23, *p* < 0.01), and an interaction between CIE exposure and perfusion time (*F*(1,61) = 4.66, *p* = 0.03, eta = 0.09, *p* < 0.01) on c‐Fos‐positive cells in the PL mPFC (Figure 2E). Post hoc comparisons revealed that rats with CIE exposure in the delayed group had a significantly greater number of c‐Fos‐positive cells than CIE rats in the immediate perfusion group. In the IL mPFC, we did not observe any significant main effects of CIE exposure or perfusion time or an interaction between the two (Figure 2F; CIE: *F*(1,20) = 2.93, *p* = 0.10; perfusion time: *F*(1,20) = 2.34, *p* = 0.14, CIE × perfusion time: *F*(1,20) = 2.92, *p* = 0.10). Overall, the data suggest that CIE exposure resulted in greater cue‐evoked neuronal activation selectively in the PL mPFC of rats perfused with a delay of 60–90 min following the final test.

### CIE does not influence cue‐evoked c‐Fos in the NAc

**FIGURE 3:**
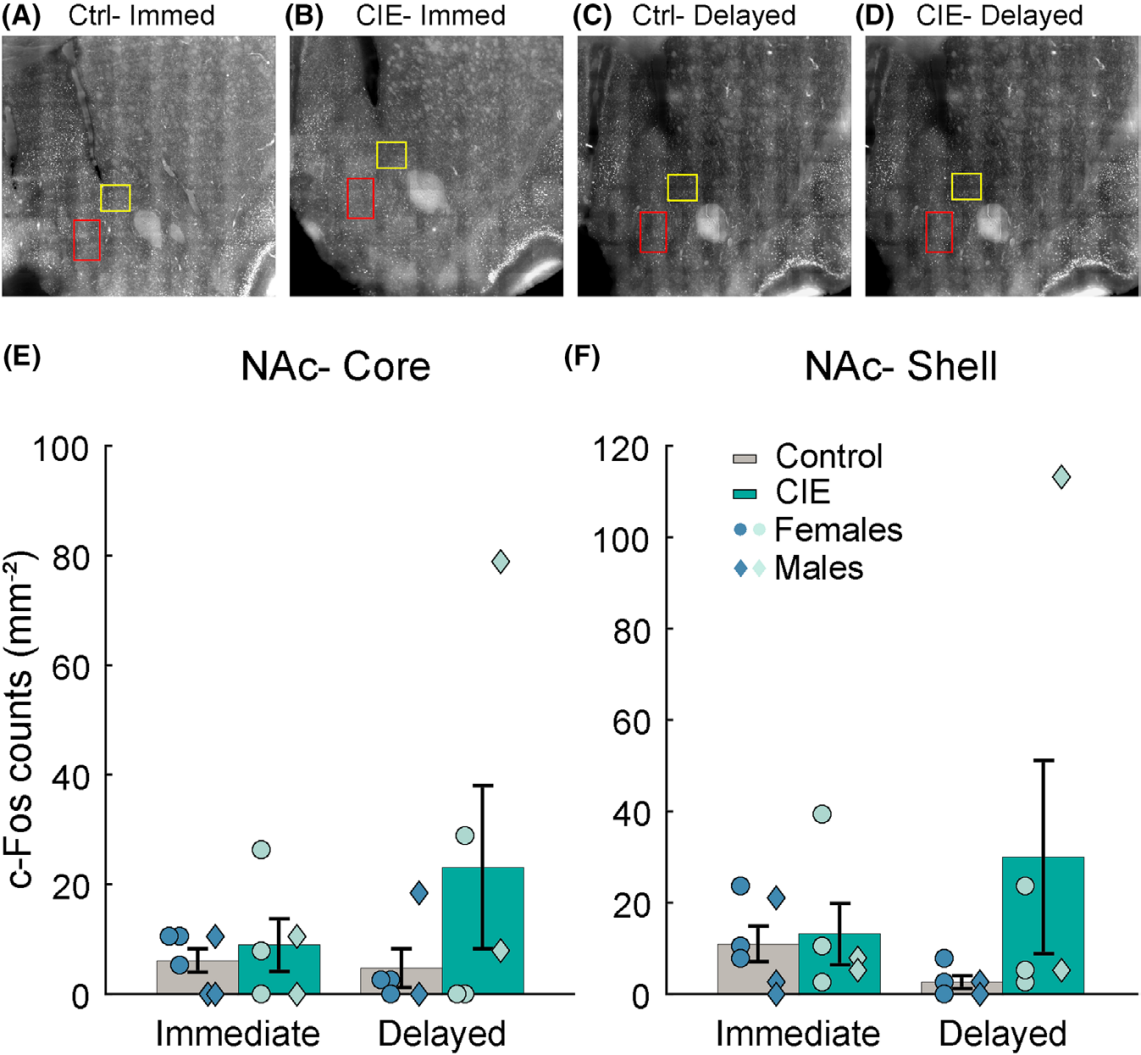
Effect of CIE vapor exposure on cue‐evoked c‐Fos in the nucleus accumbens (NAc). c‐Fos expression was analyzed in the core and shell NAc of control and CIE vapor‐exposed rats (immediate control: *N* = 6 (3 males), immediate CIE: *N* = 5 (2 males), delayed control: *N* = 5 (2 males), delayed CIE: *N* = 5 (2 males); 2 brain slices per animal). (A–D) Representative c‐Fos images, boxed region indicates the ROI used for c‐Fos quantification (core in yellow and shell in red). (E, F) No differences in cue‐evoked c‐Fos were found between control and CIE vapor‐exposed rats in the NAc core and shell. Data represented as mean ± sem. Symbols represent individual animals (circles: Females, diamonds: Males).

Next, we analyzed the effects of CIE exposure and perfusion timing on c‐Fos immunoreactivity in the NAc. In the NAc core, we did not observe any significant effect of perfusion time or interaction of CIE and perfusion time (Figure 3E, perfusion time: *F*(1,38) = 0.02, *p* = 0.88; CIE × perfusion time: *F*(1,38) = 1.26, *p* = 0.26), but we observed a trend towards a main effect of CIE (*F*(1,38) = 3.36, *p* = 0.07). Similarly, in the NAc shell, we observed a trend towards significance for a main effect of CIE (Figure 3F, *F*(1, 34) = 3.80, *p* = 0.058) but no effect of perfusion time or interaction between CIE and perfusion time (Figure 3B, *F* values ranging from 0.38 to 1.68, *p* values from 0.20 to 0.53). Our data suggest that rats with CIE vapor exposure that were perfused with a delay had more c‐Fos‐positive cells in both NAc core and shell (Figure 3E,F). However, NAc c‐Fos was highly variable, especially in CIE rats, and these trends are likely driven by outlier rats.

### Effects of CIE on cue‐evoked hippocampal c‐Fos differ by subregion

**FIGURE 4:**
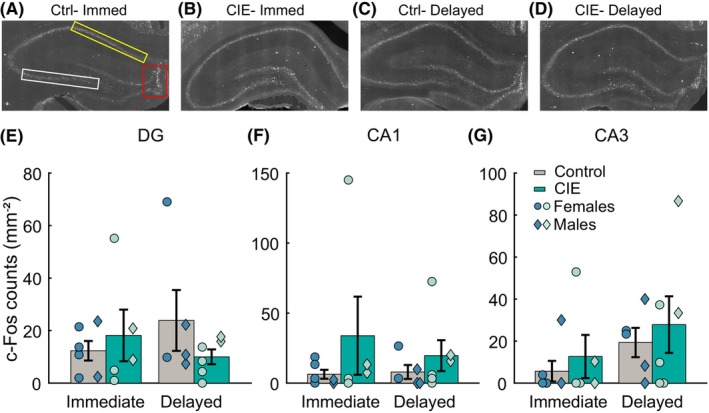
Effect of CIE vapor exposure on cue‐evoked c‐Fos in the hippocampus (HPC). c‐Fos expression was analyzed in the DG, CA1, and CA3 subdivisions of the HPC in control and CIE vapor‐exposed rats (immediate control: *N* = 6 (2 males), immediate CIE: *N* = 5 (2 males), delayed control: *N* = 5 (3 males), delayed CIE: *N* = 6 (2 males); 3 brain slices per animal). (A–D) Representative c‐Fos images, boxed region indicates the ROI used for c‐Fos quantification (DG (red), CA1 (yellow), and CA3 (white)). (E–G) No significant differences in cue‐evoked c‐Fos were found between control and CIE vapor‐exposed rats in the DG, CA1, and CA3. Data represented as mean ± sem. Circles represent individual rats. Symbols represent individual animals (circles: Females, diamonds: Males).

We next assessed the effects of CIE exposure and perfusion time on cue‐evoked c‐Fos immunoreactivity in the DG, CA1, and CA3 hippocampal subdivisions. In the DG, our analysis revealed a trend towards a significant main effect of CIE (Figure 4E, *F*(1,115) = 3.36, *p* = 0.06) but not perfusion time (Figure 4E, *F*(1,115) = 0.86, *p* = 0.35), and a significant interaction between these factors (Figure 4E, *F*(1,115) = 4.81, *p* = 0.03, eta = 0.13, *p* < 0.01), though this effect was not explained by any significant pairwise differences. The data suggested that CIE vapor exposure led to a decrease in DG c‐Fos expression in rats perfused with a delay. In the CA1 HPC subdivision, we observed no significant effect of either CIE or perfusion time or any interaction between the two (Figure 4F, *F* values ranging from 0.03 to 2.63, *p*‐values from 0.10 to 0.84). In the CA3 subdivision, our analysis revealed no significant main effects of CIE or perfusion time (Figure 4G, *F* values ranging from 1.15 to 1.82, *p* values from 0.17 to 0.28) and no interaction between CIE and perfusion time (*F*(1,115) = 1.21, *p* = 0.27). Overall, we observed different patterns of CIE vapor exposure‐mediated cue‐evoked c‐Fos expression within the hippocampal subdivisions. While CIE vapor‐exposed rats perfused with a delay had decreased cue‐evoked c‐Fos expression in the DG compared to control rats, it appeared that CIE vapor‐exposed rats in the immediate and delayed perfusion groups had elevated levels of c‐Fos in both CA1 and CA3 compared with control rats, though we did not detect any significant differences.

### Relationship between c‐Fos and behavior during the final test

**FIGURE 5:**
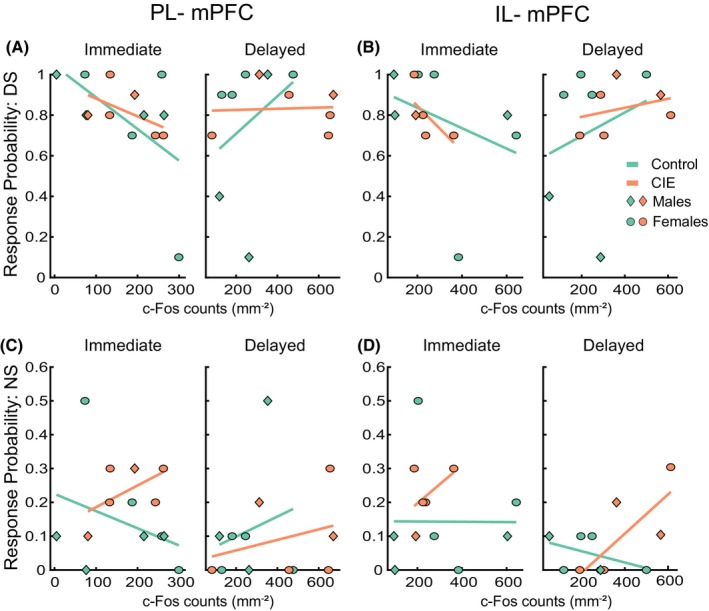
c‐Fos expression in mPFC does not predict behavioral performance. We examined whether c‐Fos expression in the PL and IL mPFC was predictive of rats' performance on the final behavioral task using LME analysis. A generalized linear model (GLM) fit was plotted to depict the relation between c‐Fos expression and response probability to the DS (A, B) and NS cues (C, D) in rats perfused either immediately following the behavioral task (Immediate) or with a delay (Delayed). Symbols represent individual rats (diamond: males, circles: females; PL mPFC: immediate control: *N* = 8 (4 males), immediate CIE: *N* = 6 (2 males), delayed control: *N* = 7 (3 males), delayed CIE: *N* = 6 (2 males); IL mPFC: immediate control: *N* = 7 (3 males), immediate CIE: *N* = 5 (1 male), delayed control: *N* = 6 (2 males), delayed CIE: *N* = 6 (2 males)).

Since the brain regions we assessed for c‐Fos immunoreactivity are implicated in cue‐elicited reward‐seeking behavior, we wanted to examine whether the c‐Fos counts, particularly in rats that were perfused immediately following the probe test, were predictive of behavioral performance on the final test. To this end, we analyzed the effects of the c‐Fos count and perfusion time on port entry probability on the final test using LME analysis. Since we did not observe a significant effect of CIE exposure on behavioral performance during the final test (Figure 1), data were pooled across CIE exposure and sex groups. We observed a trend towards significance for a main effect of c‐Fos count on the DS port entry probability in the PL mPFC (Figure 5A, *F*(1,23) = 4.26, *p* = 0.05) as well as for perfusion time (*F*(1,23) = 3.65, *p* = 0.06) along with a significant interaction between c‐Fos count and perfusion time (*F*(1,23) = 5.16, *p* = 0.03). Control rats in the immediate group that had lower c‐Fos expression had higher DS probability values, and this trend seemed to be reversed in control rats in the delayed perfusion group. However, no post hoc comparisons were significant after correction for multiple comparisons.

In the IL mPFC, we did not observe a significant main effect of both c‐Fos count and perfusion time or an interaction between the two (Figure 5B, *F*(1,20) values ranging from 1.73 to 3.03, *p* values from 0.09 to 0.20). We did not observe any significant main effects or interaction for NS port entry probability in the PL or IL mPFC (Figure 5C,D, *F* values ranging from 0.021 to 3.87, *p* values from 0.06 to 0.88).

**FIGURE 6:**
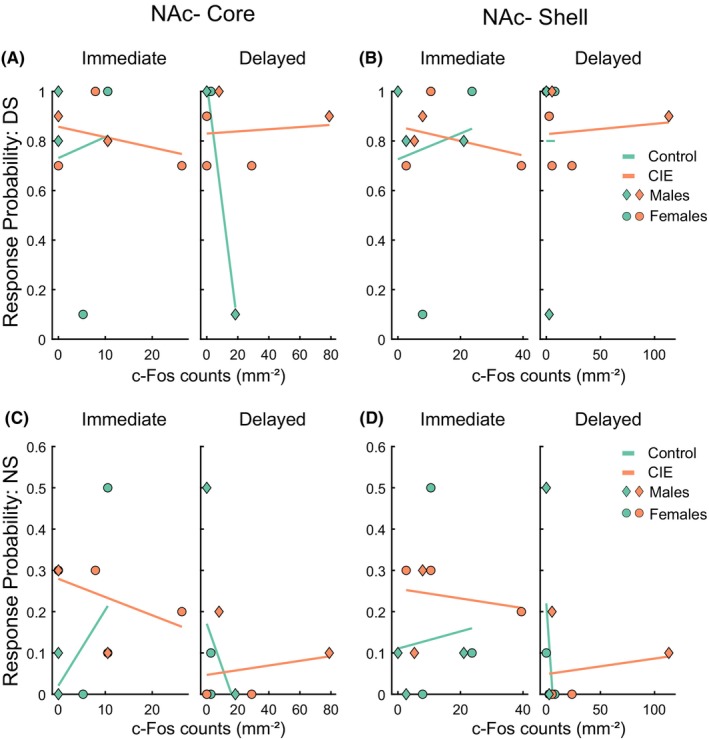
c‐Fos expression in the NAc does not predict behavioral performance. A GLM fit was plotted to depict the relation between c‐Fos expression and response probability to the DS (A, B) and NS cues (C, D) in rats perfused either immediately following the behavioral task (Immediate) or with a delay (Delayed). Symbols represent individual rats (diamond: males, circles: females; immediate control: *N* = 6 (3 males), immediate CIE: *N* = 5 (2 males), delayed control: *N* = 5 (2 males), delayed CIE: *N* = 5 (2 males)).

In the NAc, we did not observe any significant main effects of c‐Fos count or perfusion time or any interactions for both the NAc core (Figure 6A,C, *F*(1,17) values ranging from 0.003 to 0.71, *p* values from 0.41 to 0.95) and NAc shell (Figure 6B,D, *F*(1,17) values ranging from 0.0001 to 0.98, p values from 0.33 to 1.00) for both DS and NS port entry probability.

**FIGURE 7:**
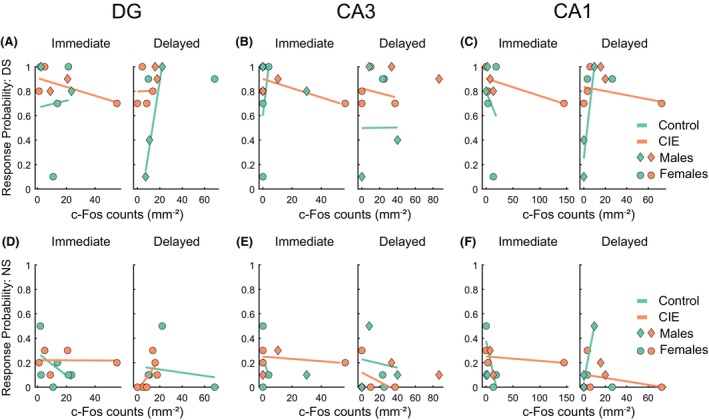
c‐Fos expression in the HPC does not predict behavioral performance. A GLM fit was plotted to depict the relation between c‐Fos expression and response probability to the DS (A–C) and NS cues (D–F) in rats perfused either immediately following the behavioral task (Immediate) or with a delay (Delayed). Symbols represent individual rats (diamond: males, circles: females; immediate control: *N* = 6 (2 males), immediate CIE: *N* = 5 (2 males), delayed control: *N* = 5 (3 males), delayed CIE: *N* = 6 (2 males)).

We observed a similar pattern in the hippocampus with no significant main effect of c‐Fos count and perfusion time, or an interaction in the DG (Figure 7A,D, *F*(1,18) values ranging from 0.17 to 1.33, *p* values from 0.26 to 0.67), CA1 (Figure 7B,E, *F*(1,18) values ranging from 0.03 to 0.59, *p* values from 0.45 to 0.85), and CA3 (Figure 7C,F, *F*(1,18) values ranging from 0.01 to 0.56, *p* values from 0.46 to 0.92) for both DS and NS port entry probability. Overall, similar to our c‐Fos results, we observed that the PL mPFC was most responsive to the effects of CIE vapor exposure, and cue‐evoked c‐Fos in the PL mPFC relates more strongly to behavioral responses to ethanol‐paired cues compared to other regions.

### Interregional c‐Fos correlations are differentially affected by CIE exposure and perfusion time

**FIGURE 8:**
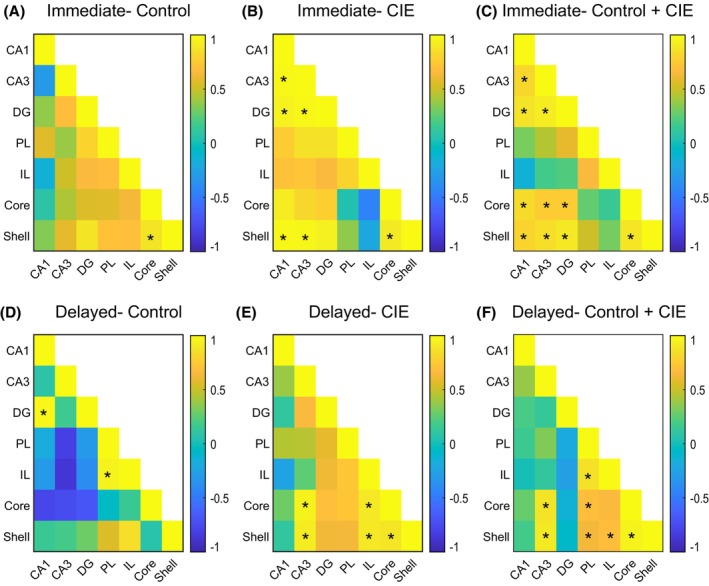
CIE vapor exposure and perfusion timing affect functional connectivity in rats. (A–F) Correlation analysis of c‐Fos expression between different brain regions. While we did not detect major changes in functional connectivity in the control animals (A vs. D), CIE vapor exposure resulted in increased number of significant correlations in rats in both immediate and delayed perfusion groups (A vs. B and D vs. E). An interaction between CIE vapor exposure and perfusion timing evoked distinct patterns of correlations within brain regions (B vs. E). (C) and (F) show that the overall pattern of correlated activity was different between rats that were perfused immediately compared to rats perfused with a delay. * represents significant correlation (*p* < 0.05). *R* values are depicted on the scale bar.

To examine the influence of CIE exposure and perfusion time on functional connectivity between the brain regions, we performed a correlation analysis on the c‐Fos counts between subregions (Figure 8). Subjects were split across the CIE exposure and perfusion time groups to assess the influence of both factors on functional connectivity (Figure 8A,B,D,E) and split just by the perfusion time to assess the overall impact of perfusion timing on functional connectivity (Figure 8C,F). In control animals that were perfused immediately, only the NAc core and NAc shell c‐Fos counts were positively correlated (Figure 8A, *R* = 0.88, *p* = 0.02) compared to rats that were perfused with a delay, where we noted a significant correlation between the DG and CA1 (Figure 8D, *R* = 0.98, *p* < 0.005) and PL and IL mPFC (Figure 8D, *R* = 0.95, *p* < 0.005). In comparison, rats with CIE vapor exposure showed correlations between c‐Fos expression in multiple brain regions (Figure 8B,E). CIE‐exposed rats that were perfused immediately showed significant positive correlations between the DG and CA1 (Figure 8B) (*R* = 0.95, *p* = 0.01), DG and CA3 (*R* = 0.98, *p* < 0.005), CA1 and CA3 (*R* = 0.98, *p* < 0.005), CA1 and NAc shell (*R* = 0.98, *p* = 0.02), CA3 and NAc shell (*R* = 0.97, *p* = 0.03), and NAc core and shell (*R* = 0.91, *p* = 0.03). CIE‐exposed rats that were perfused with a delay showed a significant correlation between c‐Fos expression in CA3 and NAc core (Figure 8E) (*R* = 0.96, *p* = 0.01), CA3 and NAc shell (*R* = 0.92, *p* = 0.03), IL and NAc core (*R* = 0.90, *p* = 0.04), IL and NAc shell (*R* = 0.90, *p* = 0.04), and NAc core and shell (*R* = 0.89, *p* < 0.005). Since the time of perfusion after stimulation can affect c‐Fos expression levels, we also examined inter‐regional correlations in c‐Fos expression with the subjects split only by the perfusion timing (Figure 8C,F). The immediate perfusion group showed a significant correlation in c‐Fos expression between the DG and CA1 (*R* = 0.87, *p* < 0.005), DG and CA3 (*R* = 0.92, *p* < 0.005), CA1 and CA3 (*R* = 0.82, *p* < 0.005), DG and NAc core (*R* = 0.75, *p* = 0.02), DG and NAc shell (*R* = 0.9, *p* < 0.005), CA1 and NAc core (*R* = 0.83, *p* = 0.01), CA1 and NAc shell (*R* = 0.8, *p* = 0.01), CA3 and NAc core (*R* = 0.75, *p* = 0.02), CA3 and NAc shell (*R* = 0.86, *p* < 0.005), PL and IL mPFC (*R* = 0.65, *p* = 0.02), and NAc core and shell (*R* = 0.89, *p* < 0.005) (Figure 8C). Rats perfused with a delay showed a significant correlation between c‐Fos expression in the CA3 and NAc core (*R* = 0.88, *p* < 0.005), CA3 and NAc shell (*R* = 0.91, *p* < 0.005), PL and IL mPFC (*R* = 0.86, *p* < 0.005), PL and NAc core (*R* = 0.68, *p* = 0.03), PL and NAc shell (*R* = 0.64, *p* = 0.04), IL and NAc core (*R* = 0.66, *p* = 0.05), IL and NAc shell (*R* = 0.68, *p* = 0.04), and NAc core and shell (*R* = 0.96, *p* < 0.005) (Figure 8F). Overall, our correlation analysis suggests that CIE vapor exposure led to differential patterns of functional correlation depending on the perfusion timing. At baseline, CIE vapor exposure‐mediated correlation included the hippocampus and NAc shell, whereas CIE vapor exposure combined with a delay in perfusion resulted in a correlation pattern involving both the NAc core and NAc shell and the PL and IL mPFC.

## DISCUSSION

Here, we examined the impact of CIE, a model of alcohol dependence, on neural activity evoked by alcohol cues. In a previous report, we found that CIE vapor exposure increased behavioral responding to cues previously paired with alcohol in rats that had access to alcohol during acute withdrawal (Carpio et al., 2022). Here, we examined the neural activation patterns across the mPFC, NAc, and hippocampus in the brains from a subset of rats used for experiment 1 in the published report (cue‐alcohol pairing followed by CIE exposure with access to ethanol during withdrawal). We used c‐Fos protein expression as a proxy for neural activity. Rats were perfused either immediately after a final behavioral test under extinction conditions or with a delay of 60–90 min, allowing us to examine the effect of the behavioral task on c‐Fos expression patterns. In the absence of a cohort that did not receive alcohol‐paired cues, we used cFos in the “immediate perfusion” group as an indicator of putative baseline level of c‐Fos depending on the rat's exposure history. This allowed us to interpret any alterations in c‐Fos levels in the “delayed perfusion” group in the context of the alcohol cues and ethanol exposure history. We found that CIE enhanced c‐Fos evoked by alcohol cues, especially in the PL mPFC. Interestingly, we also noted increased correlations between c‐Fos expression across brain regions of CIE vapor‐exposed rats, both in the immediate and delayed perfusion groups.

### Ethanol vapor inhalation and alcohol‐seeking behavior

CIE vapor inhalation has been shown to either enhance behavioral responding to previously learned alcohol–cue association (Liu & Weiss, 2002, 2003) or not affect it (Ciccocioppo et al., 2003; Eisenhardt et al., 2015). We showed in a previous report that CIE vapor inhalation enhanced behavioral responding to previously learned cue–alcohol pairing when the rats had access to ethanol during acute withdrawal (Figure 2B, Carpio et al., 2022). We used the same cohort of rats (CIE vapor inhalation and ethanol access during withdrawal) in this study and put them through another probe test before perfusion. In contrast to the previous probe test, we did not find any significant main effect of CIE exposure, though we did observe an interaction between CIE and sex. This interaction appears to be driven by elevated responding in CIE males but not females, which could suggest that the impact of CIE exposure on behavioral responding to alcohol cues is longer lasting in males than females. The difference in the impact of CIE exposure in these two behavioral tests could be explained by the long duration between the CIE exposure and final testing, which was carried out approximately 3 weeks after the completion of CIE exposure, or by repeated testing under extinction conditions. In the future, limited testing under extinction conditions with a shorter delay between completion of CIE exposure and testing might yield different insights.

### Cue‐evoked prelimbic c‐Fos is potentiated by a history of ethanol vapor inhalation and predicts alcohol‐seeking behavior

Here, we observed a significant increase in c‐Fos expression levels in the PL subdivision of the mPFC in rats exposed to CIE ethanol vapor inhalation and perfused with a delay. We interpret differences in c‐Fos between the immediate and delayed perfusion groups to be related to the impact of alcohol cues on c‐Fos expression. While a previous study found c‐Fos expression in the PL to be reduced at 74 h and 7 days of withdrawal from CIE (Smith et al., 2020), this was in the absence of exposure to alcohol cues. Our results are consistent with prior work in humans showing increased activity in the mPFC in response to alcohol‐related cues in abstinent alcoholics (Grüsser et al., 2004) and increased activation in the mPFC in response to visual cues associated with the “preferred” alcoholic beverage in subjects with AUD (Kirsch et al., 2024). When we assessed the relationship between c‐Fos levels and behavioral responding during the final test, we only observed a trend towards a main effect of PL mPFC c‐Fos on port entry probability during the alcohol DS cue. This is consistent with work showing activation of the mPFC in response to the ethanol‐paired cue when tested under extinction conditions (Groblewski et al., 2012), and a reduction in port entries in response to an ethanol cue following PL mPFC inactivation when tested under extinction conditions (Khoo et al., 2019).

### Ethanol vapor inhalation produces more variable increases in cue‐evoked NAc c‐Fos

When we examined c‐Fos in the NAc, we found interactions between CIE and perfusion time in both the shell and core that appeared to be explained by increased cue‐evoked c‐Fos in rats that were perfused with a delay, though this was not supported by any significant pairwise comparisons. Prior work has implicated both the core and shell of the NAc in cue‐elicited alcohol seeking, though the NAc core is implicated in cue‐triggered alcohol seeking irrespective of the context, while the shell NAc mediates context‐dependent alcohol seeking (Millan et al., 2015; Valyear et al., 2020). Cue‐induced reinstatement of alcohol seeking also results in elevated c‐Fos in NAc shell and core (Jupp et al., 2011). Our work extends these findings by indicating that CIE vapor exposure may potentiate these cue effects in the NAc. In contrast, in the absence of alcohol cues, CIE vapor exposure may reduce NAc signaling; CIE vapor exposure has been shown to reduce ex vivo dopaminergic transmission in the NAc of mice at both 0 and 72 h of abstinence (Karkhanis et al., 2015).

### Hippocampal c‐Fos differs by subregion, ethanol history and perfusion timing

The hippocampus is thought to contribute to drug addiction and dependence by encoding the context and cues associated with drug seeking (Kilts et al., 2001; Volkow et al., 2004). Ethanol‐predictive cues have been shown to lead to increased c‐Fos, particularly in the CA1 and CA3 subdivisions in a model of cue‐induced reinstatement (Dayas et al., 2007; Zhao et al., 2006). Previous studies have reported dynamic changes in c‐Fos expression in the hippocampus with CIE vapor exposure, withdrawal, and reaccess to ethanol across all subdivisions (Roland et al., 2023) and in the CA1 region (Smith et al., 2020). Prolonged alcohol exposure including via CIE vapor exposure has been shown to inhibit long‐term potentiation in the CA1 subdivision of the hippocampus (Roberto et al., 2002) and to produce changes at the cellular, synaptic, and molecular level such as reduction in granule cell and dendritic spine remodeling in the DG, CA1, and CA3 subdivisions, and alterations in signaling cascades involving MAPK‐CREB and ERK1/2 (Kutlu & Gould, 2016; Mira et al., 2020; Staples et al., 2015). While we observed elevated cue‐evoked c‐Fos in CA1 in controls, consistent with the prior findings, we did not observe any evidence of cue‐evoked c‐Fos in CA3. We also noted that the impact of CIE depended on both the hippocampal subregion and perfusion timing. We observed reduced DG c‐Fos expression in CIE rats perfused after a delay and potentiated CA1 and CA3 c‐Fos in CIE rats more generally. Further studies with larger sample sizes will help in ascertaining the precise nature of changes occurring within the hippocampus as a result of CIE vapor exposure and ethanol‐paired cues, and to uncover any potential sex differences.

### Ethanol vapor inhalation alters and enhances interregional c‐Fos correlations

To understand whether CIE vapor exposure had any effect on the functional connectivity between the brain regions assessed, we assessed correlations between c‐Fos expression in each region. In control animals, we did not observe many significant correlations between c‐Fos in different brain regions, irrespective of the perfusion timing. However, CIE increased the number of correlations in both perfusion groups. This finding is consistent with a decrease in the modularity of the brain that has previously been reported in rodents exposed to CIE (Kimbrough et al., 2020). Perfusion timing resulted in differing patterns of correlation, suggesting differential recruitment of neural circuits influenced by ethanol‐paired cues. CIE vapor‐exposed rats that were perfused immediately after the final behavior showed correlated c‐Fos activity within the hippocampal subdivisions and between the CA1 and CA3 and NAc shell. In rats with CIE vapor exposure that were perfused with a delay, the intra‐hippocampal correlation was lost, and instead, we saw a pattern of correlation between NAc core and shell with CA3 and IL mPFC. This shift from a hippocampus‐heavy correlation pattern to an accumbens‐heavy correlation pattern might be reflective of a cue‐mediated recruitment of neural circuits. Since the final behavioral task involved previously learned cue–ethanol associations, a lack of sufficient novelty‐based stimulation for the hippocampus may result in loss of coordinated activity both within the hippocampus and between the hippocampus and other regions. The NAc and mPFC are known to process motivational salience of reward cues and mediate goal‐directed behavior (Day & Carelli, 2007; Van Den Oever et al., 2010), and the ethanol‐paired cues in the final behavioral task probably recruit these circuits, with the correlation being observable only after sufficient time was allowed for cue‐evoked c‐Fos expression to occur.

To further understand the impact of ethanol‐paired cues on neural activity patterns, we pooled animals across the CIE vapor exposure conditions and examined correlations of c‐Fos expression between brain regions according to perfusion timing. In rats that were perfused immediately following the final behavioral test, we observed correlated activity within the hippocampal subdivisions and between the hippocampus and NAc. Rats perfused with a delay following the behavioral test showed a different pattern of correlated activity, with the majority of correlations occurring between the mPFC and NAc subdivisions and with only the CA3 subdivision being correlated with the NAc. This observation of coordinated activity changes shifting to NAc and mPFC following a cue‐elicited behavioral task suggests that the ethanol‐based cues might recruit the NAc and mPFC owing to their role in representing the motivational value of the cue. This is consistent with existing evidence that both NAc and the mPFC are involved in cue‐induced alcohol seeking and cue‐induced reinstatement of alcohol seeking (Chaudhri et al., 2010; Dayas et al., 2007; Keistler et al., 2017; Sciascia et al., 2015).

### Caveats and limitations

A key limitation of the present study is the small sample size for the c‐Fos analysis, especially for the analysis of potentially sex‐biased effects. This is important to note because despite there being evidence of sex differences in both ethanol consumption and ethanol‐paired cue reactivity, mechanisms underlying these sex differences remain poorly investigated (Barker & Taylor, 2019; Carpio et al., 2022; Morales et al., 2015; Xie et al., 2019). The inclusion of two different perfusion time points in our design led to a further reduction of sample size which limited our ability to draw meaningful inferences from c‐Fos expression in male versus female rats. Future studies with larger sample sizes including both sexes will allow for better understanding of the impact of sex on the effects of CIE vapor exposure on alcohol cue reactivity.

## CONCLUSIONS

Here, we found that CIE vapor exposure enhanced cue‐evoked c‐Fos, especially in the PL mPFC. This observation supports previous findings that implicate the mPFC in the behavioral effects of alcohol cues and withdrawal. We then assessed interregional c‐Fos correlations and found differing patterns of correlations between CIE vapor‐exposed rats perfused either immediately or with a time delay. Our results suggest that alcohol‐paired cues can lead to recruitment of different neuronal circuits, and that this could be further influenced by a history of CIE. Future studies with larger sample sizes with both male and female subjects and including more brain areas known to be involved in alcohol‐paired cue responsivity will lead to a better understanding of the neural mechanism underlying CIE vapor exposure effects on alcohol‐paired cue‐elicited behavior.

## CONFLICT OF INTEREST STATEMENT

The authors have no conflict of interest to declare.

## Data Availability

The data that support the findings of this study are available from the corresponding author upon reasonable request.
